# Case report: A case of blood culture-negative *Bartonella quintana* endocarditis: blood mNGS is an efficient method for early diagnosis

**DOI:** 10.3389/fmed.2024.1449637

**Published:** 2024-11-11

**Authors:** Jun-fan Pu, Yan-ling Zhou, Min Deng, Jing Wu

**Affiliations:** ^1^Department of Infectious Disease, The People’s Hospital of Dazu District, Chongqing, China; ^2^The First Clinical College, Chongqing Medical University, Chongqing, China

**Keywords:** *Bartonella quintana*, blood culture-negative, metagenomic nextgeneration sequencing, infective endocarditis, cardiac failure

## Abstract

*Bartonella quintana* is one of the main causes of blood culture-negative endocarditis, and routine blood culture and serological methods are difficult to achieve early diagnosis. We report a case of blood culture-negative *Bartonella quintana* endocarditis from southwestern Chongqing. The patient was a 67-year-old male scavenger who presented with heart failure without fever as the main clinical manifestation upon admission. He stated having had contact with stray cats in the past 2 months. The combination of clinical symptoms, echocardiography, and blood mNGS testing confirmed the infection of *Bartonella quintana*.

## 1 Case presentation

A 67-year-old male scavenger from a rural area in western Chongqing presented to the Dazu District People’s Hospital 3 weeks ago due to worsening edema, chest tightness, shortness of breath, and chest compression during daily activities. The patient had a history of bilateral lower extremity edema for 10 years without obvious inducing factors. The edema was symmetrical and pitting, which usually improved after rest. The patient denied any history of chronic diseases such as diabetes or hypertension, as well as any history of infectious diseases. Notably, the patient reported adopting a stray cat 2 months ago, and the working and living environmental hygiene conditions were poor for engaging in scavenging for a long time.

The patient presented to the outpatient clinic on October 11, 2023. The patient exhibited no fever, and all vital signs were within the normal range. Cardiac auscultation and bilateral lung auscultation revealed no abnormalities. Abdominal examination showed no tenderness, rebound tenderness or muscular tension. Preliminary outpatient test results indicated that the patient’s albumin level was 31.60 g/L (reference range: 40–55 g/L) and urine protein (+++). The electrocardiogram (ECG) showed sinus tachycardia and a complete left bundle branch block. The preliminary outpatient diagnosis was “edema of unknown etiology, possibly heart failure or chronic glomerulonephritis?”

**Figure 1:**
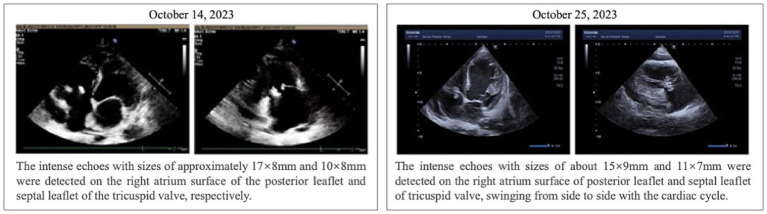
Results of echocardiography in patients.

**Figure 2:**
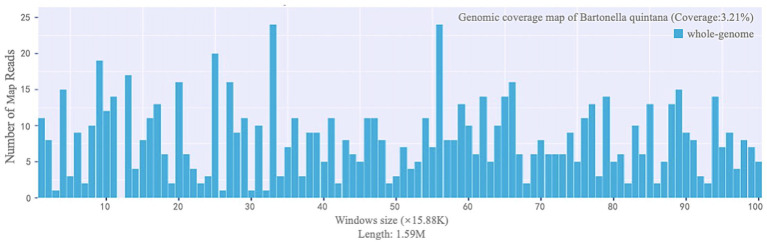
Genome coverage map of *Bartonella quintana*.

The patient was hospitalized on October 14, 2023, and relevant examinations were promptly completed upon admission. The albumin level was 28.60 g/L, and combined with the outpatient finding of urine protein (+++), hypoalbuminemia due to proteinuria was considered. The amino-terminal pro-B-type natriuretic peptide (NT-proBNP) level was elevated at 7253.23 pg./mL (reference range: 0–900 pg./mL), and the D-dimer level was 4.32 ug/ml (reference range: 0.00–1.00 ug/ml), suggesting heart failure. The high-sensitivity C-reactive protein (hs-CRP) level was 5.55 mg/L (reference range: 0–3.3 mg/L), the white blood cell count was 3.18 × 109/L (reference range: 3.5–9.5 × 109/L), the red blood cell count was 3.59 × 1,012/L (reference range: 4.30–5.80 × 1,012/L), and the hemoglobin level was 105.00 g/L (reference range: 130–175 g/L), indicating the presence of infection and anemia. The patient’s echocardiogram revealed vegetation attached to the tricuspid valve (17 × 8 mm, 10 × 8 mm) and mild regurgitation of both the mitral and tricuspid valves (Figure 1). The electrocardiogram indicated sinus tachycardia. During this period, two blood cultures were arranged for the patient, both of which were negative, making it impossible to determine the source of infection. Blood mNGS examination was performed (Instrument: MataCAPTM. Method: Probe capture high-throughput sequencing. Database: Refer to CARD, VFDB and other databases), the results suggested an infection of *Bartonella quintana* (sequence number: 1196, relative abundance: 67.16%, confidence: 99%, Genome coverage: 3.21%) (Table 1 and Figure 2).

**Table 1:** Results of high-throughput sequencing of pathogen microorganism metagenome.

Special pathogens (Mycobacterium, Mycoplasma, Chlamydia, Rickettsia, Spirochaete)						
Genus				Species		
Gram’ stain	Name	Reads	Relative abundance	Name	Reads	Confidence
G-	Bartonella	1,197	67.16%	*Bartonella quintana*	1,196	99%

Upon admission, the nephrologist temporarily prescribed sacubitril/valsartan and intravenous meglumine adenosine cyclophosphate to improve cardiac function, along with furosemide combined with spironolactone for diuretic treatment. During hospitalization, auxiliary examinations indicated elevated infection markers, and echocardiography suggested valvular vegetation, leading to a consideration of infectious endocarditis. Therefore, the patient was transferred to the infectious disease department of our hospital for further treatment on October 17, 2023. Since the patient had no symptoms such as fever or bone pain throughout the hospital stay and there was no obvious etiological basis, the infectious disease physician empirically administered an intravenous infusion of meropenem 1 g q8h combined with vancomycin 1 g q12h for anti-infection treatment. While undergoing treatment, blood mNGS testing was performed, which revealed *Bartonella quintana*. Based on the patient’s clinical manifestations, echocardiography, and other examinations, a diagnosis of *Bartonella quintana* endocarditis was confirmed.

Following the diagnosis confirmation, the patient’s medication was adjusted in alignment with the “Thermal Diseases: Sanford Antimicrobial Therapy Guidelines (50th edition).” The prescribed treatment regimen consisted of doxycycline 0.1 g orally every 12 h, complemented by intravenous infusions of rifampin 0.3 g administered every 12 h. After a week of this treatment, a reexamination revealed normalized infection indicators, and the echocardiogram demonstrated a notable decrease in valvular vegetation compared to previous observations (15 × 9 mm, 11 × 7 mm) (Figure 1). Additionally, the patient reported significant relief from symptoms such as fatigue, shortness of breath, and bilateral lower extremity edema. Throughout this period, the attending physician consistently recommended valve replacement surgery as a definitive treatment option. However, the patient and their family declined surgical intervention and provided signed informed consent to forgo surgical treatment. At the patient’s request, they were discharged on October 30, 2023. As part of the discharge instructions, the patient was prescribed a continued course of oral doxycycline (0.1 g per dose, twice daily for four weeks) to complete the infectious treatment course. Furthermore, the doctor advised a follow-up echocardiogram within three weeks to assess the progress of the valvular vegetation and reconsider surgical options if necessary.

### 1.1 Reads

This refers to the number of specifically measured fragments belonging to a particular microbial species. Typically, this represents the number of net reads obtained after the raw sequence data have been filtered to remove linkers and low-quality reads. A higher reads indicates a greater likelihood of the presence of that microorganism in the sample.

### 1.2 Relative abundance

This refers to the distribution proportion of sequences belonging to a particular microbial species within its corresponding broad taxonomic category (which can be divided into five groups: bacteria, fungi, viruses, parasites, and special pathogens), after excluding host sequences. A higher abundance indicates a higher proportion of that species.

### 1.3 Confidence

This is a measure of the reliability of the presence of a microorganism in the sample, based on factors such as the number of sequence alignments, genome size, and genome conservation.

### 1.4 Genome coverage

This refers to the percentage of sequenced bases that reach a given depth within the entire genome or the target region.

### 1.5 Number of mapped reads

It indicates depth and refers to the number of times a particular sequence on the microbial genome is detected. Parts that do not reach the given depth are referred to as blind gaps.

### 1.6 Window size

It is a commonly used strategy in genomic data visualization analysis, where a continuous genomic sequence intended for analysis and comparison is divided into a series of segments of equal or nearly equal length. The more uniform the distribution of the graph along the horizontal axis, the more reliable the inspection results.

## 2 Discussion

*Bartonella quintana*, a fastidious Gram-negative bacterium belonging to the genus Bartonella, is characterized by slow growth, facultative intracellular parasitism, and a high dependence on oxidized hemoglobin (1). The genus Bartonella is considered one of the common etiologies of blood culture-negative endocarditis, accounting for 2–40% of such cases, with *Bartonella quintana* being the predominant species, followed by *Bartonella henselae* (2, 3). *Bartonella quintana* parasitizes on human lice, reproduces in their intestinal cavity, and enters the human body through damaged skin and conjunctiva via lice feces, triggering infection. Outbreaks often occur among individuals with low immunity and poor personal hygiene conditions (3, 4). This bacterium was once the primary pathogenic agent of “trench fever” during World War I, and cat scratches or exposure to cat fleas are among the risk factors for this disease (5).

The clinical manifestations of Bartonella endocarditis are atypical, which increases the difficulty of diagnosing the disease. We reviewed global case reports on *Bartonella quintana* endocarditis and found that heart failure is one of the main manifestations of *Bartonella quintana* endocarditis, while fever is often not the primary symptom of the disease (6–8). A report from Spain included 8 confirmed cases of *Bartonella quintana* endocarditis, with 87.5% of patients exhibiting heart failure and only 37.5% exhibiting fever (6). A report from Ethiopia involved 5 confirmed cases of Bartonella endocarditis in pediatric patients, with 3 cases exhibiting heart failure and none of the cases exhibiting fever (7). In terms of biochemical indicators, a slight increase in C-reactive protein (CRP) is a highly probable indicator. Patients with *Bartonella quintana* endocarditis reported in Ethiopia, Guinea, and other regions all showed elevated CRP levels (7, 9). An eight-year study in the United States pointed out that most patients with Bartonella endocarditis had slightly elevated CRP levels (10). Sarsiat et al. (11) included 12 cases of endocarditis caused by *Bartonella quintana* in the southwestern Indian Ocean and noted that all patients exhibited high CRP concentrations (11). In this case, the patient’s main symptom was heart failure, and there was no fever during the consultation and hospitalization, but there was an increase in CRP, which is consistent with most case reports.

Clinically, a significant proportion of cases of *Bartonella quintana* endocarditis yield negative microbiological cultures, this increases the risk of delayed diagnosis and misdiagnosis due to the fastidious growth requirements and slow growth rate of Bartonella, which evade identification by conventional blood culture methods (12). Active microbial PCR detection or histopathological evidence from valve specimens can serve as the gold standard for confirming culture-negative Bartonella endocarditis, while these methods can only be performed post-surgery and are not helpful for early diagnosis and treatment of pre-surgical and surgically contraindicated patients. In this study, the patient’s two blood culture tests were negative, and the patient refused the surgeon’s recommendation for surgery. Therefore, with the patient’s consent, we performed serum mNGS testing on the fifth day of admission and received the results the following day. This allowed for early confirmation of *Bartonella quintana* infection and timely adjustment of the medication regimen. In the latest iteration of the Duke criteria, metagenomic next-generation sequencing (mNGS) has emerged as a new primary standard for identifying Bartonella species from blood (13). Its sensitivity and specificity have been rigorously validated through tests involving spiking known microorganisms into blood samples (14). The rapid turnaround time of 24–48 h for results makes mNGS an efficient, noninvasive diagnostic tool for early detection of Bartonella endocarditis.

mNGS (metagenomic next-generation sequencing) enables rapid and unbiased detection of multiple pathogenic microorganisms by performing high-throughput sequencing of the entire biome genome in clinical samples and comparing the sequences to known microbial genome sequences in a database. The basic process of mNGS in clinical microbiology laboratories is relatively complex, broadly encompassing steps such as specimen collection, transportation, nucleic acid extraction and enrichment, library preparation and sequencing, as well as bioinformatics analysis and comparison. Throughout the entire process, strict aseptic operations and effective anti-contamination measures must be adhered to in order to ensure the accuracy of sequencing results and database comparison outcomes. Despite its promise, there are currently limited reports on the clinical application of mNGS in diagnosing endocarditis. In a prospective study led by Zeng et al. (15) involving 99 patients with infective endocarditis (IE), data from the study supports valvular mNGS as the preferred diagnostic method during the perioperative phase of cardiac surgery, which holds particular significance for patients with negative blood cultures or those suspected of IE (15). Another prospective study including 79 LSIE patients, their results revealed that venous/arterial blood-based mNGS displayed a significantly higher positive rate than blood/valve culture and demonstrates that the combination of blood mNGS and blood culture offers an effective means of identifying pathogenic microorganisms. This integrated approach facilitates timely diagnosis and treatment and provides invaluable insights for optimizing antibiotic therapy in high-risk patients with infective endocarditis (16). The sample collected in this study was a blood sample, which, compared to mNGS testing of the valve tissue samples, offers the advantages of being non-invasive, rapid to collect, and requiring no de-humanization processing. However, clinical studies have indicated that the detection rate of blood samples is slightly lower than that of valve tissue samples, albeit still higher than that of blood cultures and valve cultures (16).

The interpretation of mNGS (metagenomic next-generation sequencing) test results is complex, requiring comprehensive consideration of multiple factors including reads, relative abundance, and genome coverage. In this study, the reads of *Bartonella quintana* detected by mNGS in the sample was 1,196, which is relatively high. In terms of relative abundance, Bartonella, as a genus, accounted for 66.7% of the abundance within the broad category of special pathogens in the sample. Regarding genome coverage, the whole-genome coverage of *Bartonella quintana* in the sample was 3.21%. Combined with the genome coverage map, it was found that the reads was relatively evenly distributed across different window sizes, with almost no blind gaps, indicating reliable test results (Table 1, Figure 2).

Surgical excision of vegetation combined with aortic valve replacement surgery represents the optimal treatment approach for the eradication of Bartonella endocarditis. Currently, most case reports on *Bartonella quintana* have undergone valve replacement surgery. In this case, the patient refused surgery for personal reasons, despite repeated attempts to persuade them otherwise. As a result, the physician maintained a combination therapy of doxycycline and rifampin for anti-infective treatment. After 1 week of treatment, the size of the valve vegetation decreased, and the patient’s symptoms of fatigue, shortness of breath, and bilateral lower extremity edema were significantly relieved. The patient strongly requested discharge. To control infection indicators and prevent further deterioration of the condition, the physician prescribed continued oral administration of doxycycline as an outpatient treatment, and increased the frequency of follow-up visits, requiring the patient to return for review within 3 weeks. Depending on the results, the patient and their family were advised to consider surgical treatment.

## 3 Conclusion

The diagnosis of *Bartonella quintana* can be challenging, its clinical manifestations are atypical, and conventional blood culture and serological methods suffer from long processing times and low sensitivity. Blood mNGS testing offers advantages of high sensitivity, efficiency, and non-invasiveness. We believe that combining patient clinical features with echocardiography and early use of mNGS to test blood samples can facilitate early diagnosis and targeted antimicrobial therapy, especially in cases where valve specimens cannot be obtained.

## Data Availability

The original contributions presented in the study are included in the article/supplementary material, further inquiries can be directed to the corresponding authors.
